# Trop2: Jack of All Trades, Master of None

**DOI:** 10.3390/cancers12113328

**Published:** 2020-11-11

**Authors:** Sára Lenárt, Peter Lenárt, Jan Šmarda, Ján Remšík, Karel Souček, Petr Beneš

**Affiliations:** 1 Department of Experimental Biology, Faculty of Science, Masaryk University, 625 00 Brno, Czech Republic; 405249@mail.muni.cz (S.L.); 408720@mail.muni.cz (P.L.); smarda@sci.muni.cz (J.Š.); ksoucek@ibp.cz (K.S.); 2 Research Centre for Toxic Compounds in the Environment, Faculty of Science, Masaryk University, 625 00 Brno, Czech Republic; 3 Human Oncology & Pathogenesis Program, Memorial Sloan Kettering Cancer Center, New York, NY 10065, USA; remsikj@mskcc.org; 4 Department of Cytokinetics, Institute of Biophysics of the Czech Academy of Sciences, 612 65 Brno, Czech Republic; 5 Center of Biomolecular and Cellular Engineering, International Clinical Research Center, St. Anne’s University Hospital, 656 91 Brno, Czech Republic

**Keywords:** Trop2, TACSTD2, cancer, proliferation, metastases, epithelial-to-mesenchymal transition, therapy

## Abstract

**Simple Summary:**

Metastatic progression represents a major clinical challenge and remains the leading cause of death in cancer patients. Identification of suitable therapeutic targets, followed by a rational design of effective drug agents and their long-term, extensive pre-clinical and clinical testing is one of the key goals in cancer research. Trophoblast cell surface antigen 2 (Trop2) was first described as a protein highly expressed on the surface of trophoblast cells in 1981. Just recently, nearly 40 years after this discovery, sacituzumab govitecan, a Trop2-targeting antibody drug conjugate, has been granted an accelerated approval for therapy of metastatic triple-negative breast cancer, opening a new chapter of such successful story. The aim of this review is to summarize the current knowledge about Trop2 function in healthy tissue and pathology, with a special focus on its still controversial role in plasticity and heterogeneity during cancer progression. We further discuss the development and potential of Trop2-targeted therapy.

**Abstract:**

Trophoblast cell surface antigen 2 (Trop2) is a widely expressed glycoprotein and an epithelial cell adhesion molecule (EpCAM) family member. Although initially identified as a transmembrane protein, other subcellular localizations and processed forms were described. Its congenital mutations cause a gelatinous drop-like corneal dystrophy, a disease characterized by loss of barrier function in corneal epithelial cells. Trop2 is considered a stem cell marker and its expression associates with regenerative capacity in various tissues. Trop2 overexpression was described in tumors of different origins; however, functional studies revealed both oncogenic and tumor suppressor roles. Nevertheless, therapeutic potential of Trop2 was recognized and clinical studies with drug–antibody conjugates have been initiated in various cancer types. One of these agents, sacituzumab govitecan, has been recently granted an accelerated approval for therapy of metastatic triple-negative breast cancer. In this article, we review the current knowledge about the yet controversial function of Trop2 in homeostasis and pathology.

## 1. Gene and Protein

Trophoblast cell surface antigen 2 (Trop2) was first described in 1981 as a protein highly expressed on the surface of trophoblast cells [1]. It is also known as EGP-1, M1S1, and GA733-1 [2]. Trop2 is encoded by the *TACSTD2* (tumor-associated calcium signal transducer 2) gene, which is a member of the *TACSTD* gene family [3,4]. The second member of this family, *TACSTD1* (also known as *EpCAM*), encodes epithelial cell adhesion molecule (EpCAM). Trop2 and EpCAM share 49% identity and 67% similarity of amino acid sequence with highly similar distribution of hydrophilic and hydrophobic residues [5,6]. The results of sequence analysis suggest that in the evolution of *TACSTD* gene family exon shuffling occurred, since some exons of *TACSTD* genes are homologous to exons of genes coding for thyroglobulin, HLA-DR-associated invariant chain, and probably nidogen [7]. All nine exons of *TACSTD1* are represented in the intronless *TACSTD2*, suggesting that it was formed by retroposition via an mRNA intermediate. This retroposition preceded the divergence of avian and mammalian lineages, thus *TACSTD* genes are more than 300 million years old. Both *TACSTD1* and *TACSTD2* genes are highly conserved across species [7,8]. Examples of species with *TACSTD2* include mice, chimpanzees, goats, sheep, chickens, and many others according to the Uniprot search [9]. In humans, the intronless *TACSTD2* gene is located on chromosome 1 in 1p32 locus [3].

**Figure 1:**
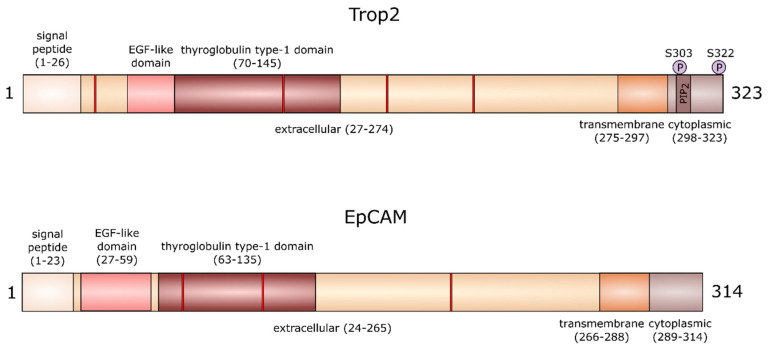
The structure of trophoblast cell surface antigen 2 (Trop2) and epithelial cell adhesion molecule (EpCAM) proteins. The red bands represent N-linked glycosylation sites at positions 33, 120, 168, and 208 in Trop2 and positions 74, 111, and 198 in EpCAM.

Human Trop2 is a 35 kDa large transmembrane protein with four N-linked glycosylation sites, consisting of 323 amino acids [4]. The extracellular domain at N-terminus starts with a 26-amino-acid-long hydrophobic signal peptide [4]. The rest of the extracellular domain is formed by 248 amino acids and contains 12 cysteine residues, epidermal growth factor (EGF)-like domain, and thyroglobulin motif. The short transmembrane domain is made of 23 amino acids and the cytoplasmic tail of 26 amino acids. The cytoplasmic domain contains a motif homologous to the phosphatidylinositol-4,5-bisphosphate (PIP_2_)-binding sequence of gelsolin [8], a protein that affects actin filament organization. In this motif, there is a serine at position 303, which may be phosphorylated by protein kinase C (PKC) (Figure 1) [10]. This phosphorylation leads to conformational changes and affects the accessibility of functionally important regions of its cytoplasmic tail [11]. It has been shown that Trop2 extracellular domains can form dimers [12]. Using molecular dynamic simulations, Pavšič et al. later demonstrated that the Trop2 dimerization interface extends to the transmembrane part [11]. However, the effect of dimerization on Trop2 function has not yet been studied in detail.

A recent study shows that serine residue at position 322 may also be phosphorylated by PKC [13], which was predicted earlier for cytoplasmic Trop2 [14]. This phosphorylation is critical for the highly motile phenotype of colorectal carcinoma cells [13]. Interestingly, there are no serine residues equivalent to Trop2 S303 and S322 in EpCAM, suggesting a differences in signaling properties of cytoplasmic domains of both proteins [11]. Both proteins also differ in the number of glycosylation sites (Figure 1). Whether these differences in the structure of Trop2 and EpCAM are reflected in their function in health and disease remains to be determined.

## 2. Trop2 in Healthy Tissue and Development

Trop2 expression was detected in healthy epithelial cells of many organs including respiratory tract, cervix, endometrium, fallopian tubes, placenta, seminal vesicles, thymus, vagina, esophagus, skin, tonsils, cornea, breast, kidney, pancreas, prostate, salivary glands, uterus, lung, stomach, colorectum, and bile duct epithelium of the liver [15,16]. Besides the above-mentioned organs, Trop2 is also expressed in lungs [17,18], intestines [19], stomach [20], bladder [21], and kidneys [22] during embryonal and fetal development. Trop2 protein was also detected in granule cells in all layers of the developing cerebellum, particularly in postmitotic cells, suggesting its function in regulation of cell migration [23]. In the damaged adult stomach, Trop2 is re-expressed and might be associated with regeneration processes [20]. The pro-regenerative potential of Trop2-positive cells was also described in the in vivo model of murine endometrial regeneration [24], and Trop2-positive cortical bone-derived stem cells hold superior pro-regenerative effect after myocardial infarction in mice [25].

Trop2 is also considered a stem/progenitor cell marker. High expression of this protein has been found in murine mesenchymal stem cells (mMSC). mMSC from Trop2 KO mice have prolonged cellular doubling time and impaired differentiation to adipocytes and osteoblasts [26]. Interestingly, a very recent study found overexpression of Trop2 in the in vitro model of osteoidosis and in the bone surface of patients with osteomalacia [27]. The authors of this study proposed that increased expression of Trop2 might act as a stimulator of MSC osteogenic differentiation in an attempt to improve the bone structure by osteoblasts.

Trop2 also marks the subpopulation of human and murine prostate basal cells with stem cell characteristics [28]. We have recently shown that Trop2-positive mouse basal prostate stem cells are enriched in the expression of epithelial-to-mesenchymal transition (EMT) master regulator Slug and that Slug expression further associates with increased organoid forming capacity of these cells [29]. While Trop2 expression in basal cells does not change during aging, it was found that Trop2 is significantly upregulated in the “old” prostate luminal cells when compared to adult luminal cells and that Trop2-positive cells contribute to the vast majority of luminal progenitor activity in old mouse prostate [30]. Furthermore, expression of Trop2 has been found in murine bladder progenitor cells [21], intestinal progenitor cells [19], and oval cells [31], which are considered as possible liver stem cells.

In patients with chronic obstructive pulmonary disease (COPD), Trop2 expression is significantly increased in airway basal cells, acting as the stem/progenitor cells of airway epithelium [32]. In vitro, Trop2 promotes proliferation and self-renewal of basal cells and induces an EMT-like phenotype and the release of proinflammatory cytokines. This observation suggests that Trop2 might be crucial in early airway repair abnormalities and remodeling in COPD patients. Association between Trop2 and lung cell proliferation was also reported during normal fetal lung growth in sheep and confirmed by RNA interference in rat fetal lung fibroblasts [17,33]. In kidney, however, Trop2-positive cells did not proliferate and ectopic expression of Trop2 inhibited cell migration and branching [22].

Trop2 also marks the biliary and liver progenitor cells, discovered after sequencing of EpCAM^+^ cells from adult human livers, widely considered as hepatic stem cells [34]. Intriguingly, there is a gradient of Trop2 expression in EpCAM^+^ cells, and cells with intermediate Trop2 expression exhibit the highest capacity to form organoids. Subsequent flow cytometric analysis of these organoids revealed that the populations with high, intermediate, and low or no expression of Trop2 are present again. Authors suggest that the fraction of cells with intermediate Trop2 expression contains a rare cell population with the capacity to differentiate into both hepatocytes and cholangiocytes [34].

Although the results of these studies suggest the active role of Trop2 in the regulation of stem cell proliferation, migration, and regenerative potential in various tissues, the function of Trop2 in embryonal development has not been fully understood yet. Trop2 null mice are surprisingly fully viable, fertile, and lack overt developmental defects, suggesting that Trop2 is not required for mouse embryonal development and fertility [35]. This is in striking difference to EpCAM null mouse models that display severe intestinal dysfunction [36,37,38]. The loss of intestinal tissue integrity observed in these models corresponds to signs of congenital tufting enteropathy (CTE), a disease caused by *EpCAM* mutations in humans [39]. Interestingly, while EpCAM is expressed by intestinal epithelial cells (IEC) of the developing and adult gut, TROP2 is expressed only in the former [15,19,40]. Recently, Nakato et al. showed that forced expression of Trop2 in IEC of EpCAM null mice largely prevented manifestation of CTE. However, the abnormalities in histology and physiology of intestine of these mice revealed that the function of both proteins is not equivalent [41].

These results suggest that Trop2 is a robust marker of adult stem cell populations, but its function is most likely dispensable or compensated by other, similar molecule(s). Further studies will be critical to clarify this issue.

### 2.1. Germline Mutations in Trop2 (TACSTD2) Gene

Congenital mutations in the human *TACSTD2* gene cause a gelatinous drop-like corneal dystrophy (GDLD) [42]. GDLD is a rare autosomal recessive disease that leads to the development of bilateral corneal amyloidosis and eventually blindness, with the highest prevalence in Japan. Currently, there are 32 known mutations in *TACSTD2* causing GDLD [43], and four distinct subtypes of GDLD: band keratopathy type, stromal opacity type, kumquat-like type, and typical mulberry type [44]. Since the same mutation can lead to different phenotypes, GDLD phenotypes also depend on secondary factors. The most common mutation is the substitution in the codon coding for glutamine at position 118 for a stop codon in the *TACSTD2* gene [42]. This substitution leads to the expression of truncated Trop2 that lacks the transmembrane and intracellular domains and aggregates in the perinuclear area. Such loss of function of Trop2 in the corneal cells of GDLD patients is responsible for impaired subcellular localization of tight junction-related proteins, i.e., claudins and occludins, and thickened basal membrane [45]. In the basal membranes and amyloid deposits, Takaoka et al. discovered the presence of Lactoferrin, known as a major component of tear fluid. Loss of barrier function of corneal epithelial cells allows Lactoferrin to pass through the basal membrane to the subepithelial region, resulting in the formation of amyloid deposits. It is not known why *TACSTD2* mutation displays disease phenotype specifically in the cornea, while other organs covered by stratified epithelia remain morphologically and functionally unaffected [46].

In the immortalized corneal epithelial cells in vitro, knockout of *TACSTD2* and *EpCAM* genes leads to the reduced epithelial barrier function and decreased expression and altered subcellular localization of Claudins 1 and 7 [47]. While the deletion of *TACSTD2* affects only localization of Claudin 1, the deletion of both *TACSTD2* and *EpCAM* genes adequately reflects the phenotype of GDLD corneal epithelial cells. Due to their close phylogenetic relationship, EpCAM is likely able to compensate for the loss of Trop2 function in vitro. Since EpCAM is not expressed in corneal epithelial cells in vivo, mutation of the *TACSTD2* gene alone sufficiently promotes the development of GDLD [47].

**Figure 2:**
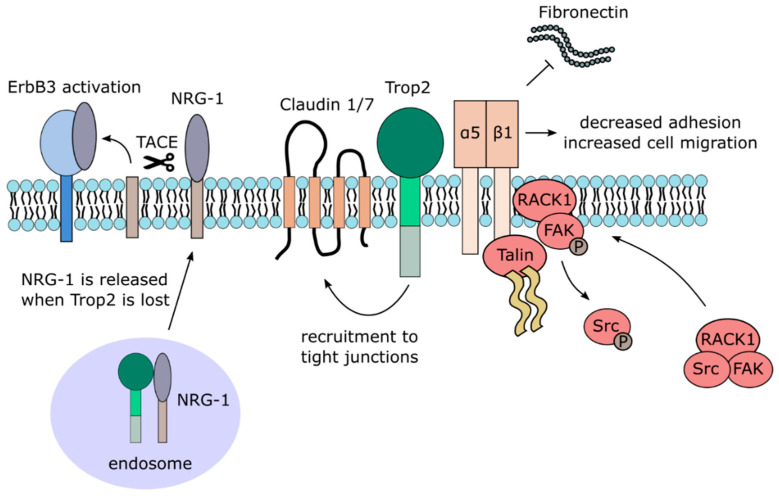
Trop2 membrane-associated interacting partners and signaling.

The causal relationship between *TACSTD2* gene mutation and GDLD suggests that Trop2 plays a critical role in the formation of tight junctions in stratified epithelia. Previous studies report the ability of Trop2 to bind to Claudins 1 and 7 [46]. The exact binding site is yet to be revealed. Presumably, Trop2 binds to claudins through their AxxxG motifs in transmembrane regions, resembling the EpCAM-Claudin 7 interaction [48]. The function of such Trop2 interaction with claudins is not entirely known yet. Nevertheless, a recent study shows that the interaction of Trop2 and Claudin 7 may lead to the recruitment of Claudin 7 to tight junctions [13]. It is known that Trop2 knockdown leads to decreased expression and altered subcellular localization of claudins and other tight junction-related proteins by modulation of their phosphorylation by PKC (Figure 2) [46,49]. The phosphorylation status of Trop2 might likely be crucial for proper localization of claudins. Phosphorylation of serine 322 causes decreased co-immunoprecipitation of Trop2 and Claudin 7, which leads to loss of Claudin 7 stability [13].

The function of Trop2 in regulation of Claudin 1 and Occludin cellular localization was also described in hepatocellular carcinoma cells and primary hepatocytes. Mechanistic studies revealed that Trop2 silencing blocked the phosphorylation of Claudin 1 and Occludin, altered their proper cellular localization and inhibited hepatitis C virus infection at the level of viral entry into hepatocytes [49]. Recently, membrane-anchored epithelial protease Matriptase was shown to cleave Trop2 and EpCAM proteins [50,51,52]. Interestingly, elimination of both proteins was necessary to reduce claudin levels in keratinocytes confirming the functional redundancy of both protein in regulation of epithelial barrier function [50].

### 2.2. Trop2 Signaling and Interaction Network

An early report published more than two decades ago first suggested that Trop2 acts as a calcium signal transducer by increasing calcium (Ca^2+^) concentration in the cytosol [53]. The study also proposed that Trop2 induces the release of calcium from internal reserves, since the signal is transferred even in the absence of extracellular calcium ions. Such calcium signal transduction function was assigned to the intracellular domain containing the PIP_2_-binding sequence [8]. However, this phenomenon has not been further investigated or independently confirmed. A more detailed, mechanistic insight into the role of Trop2 as a calcium signal transducer is thus desperately needed.

By contrast, numerous studies reported activation of Akt kinase by Trop2, mostly in cancer cell lines [54,55,56,57,58] but also in murine mesenchymal stem cells [26]. A large proteomic analysis recently identified more than 100 signaling molecules modulated by Trop2, with PTEN/PIK3CA/Akt/GSK3ß being a major activated pathway in cancer cells [59].

**Figure 3:**
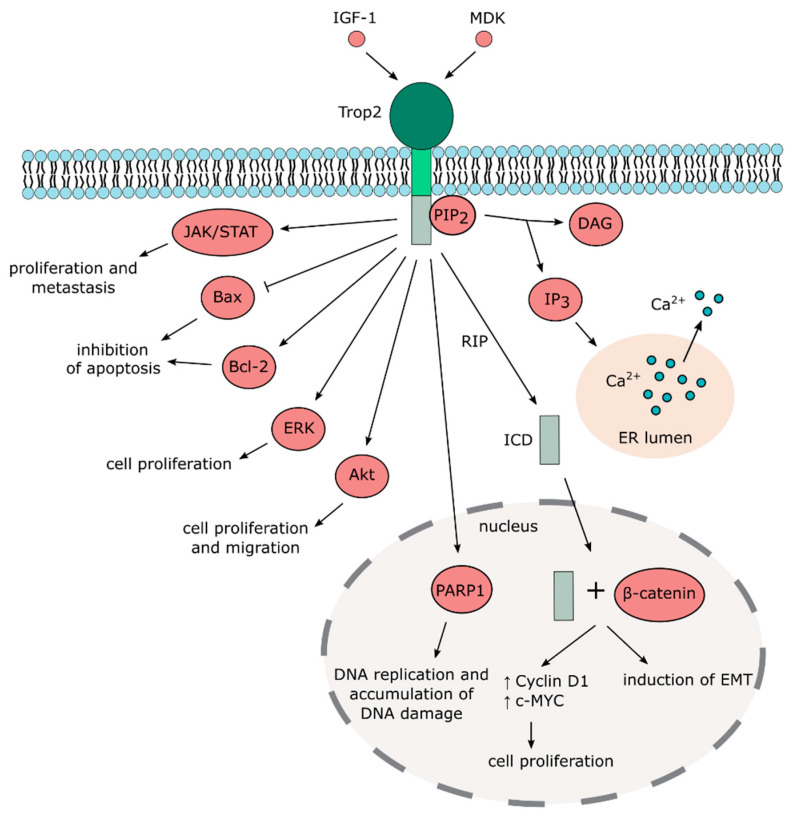
Trop2-mediated signaling pathways.

Other signaling pathways identified in this large screen that has been confirmed in different studies include MAPK/ERK [60,61,62], ErbB [63,64,65], TGFβ [66], Wnt/β-catenin [66,67], JAK/STAT [68], integrin signaling [69,70,71,72], and adherent and tight junction signaling (Figure 2 and Figure 3). It should also be noted that the downregulation of Akt and MAPK/ERK pathways by Trop2 were also described [65,73,74,75], suggesting that the effect of Trop2 may be cell context-dependent.

Despite the fact that Trop2 is considered a transmembrane protein, it can also be localized in the cytoplasm with possible functional consequences [42,65,76]. Cytoplasmic, but not membrane-bound Trop2, expression positively correlates with phospho-Akt in breast cancer specimens [59]. Stoyanova et al. reported that Trop2 undergoes regulated intramembrane proteolysis (RIP) [67]. During RIP, the extracellular domain is cleaved, whereas the intracellular domain is released and enters the nucleus, where it accumulates in complex with β-catenin that then activates transcription of downstream targets. The extracellular domain of Trop2 is cleaved by metalloproteinase tumor necrosis factor-α converting enzyme (TACE), while the intracellular domain (ICD) is cleaved predominantly by presenilin-1 but also by presenilin-2. Interestingly, Trop2 ICD has been found in human prostate cancer cells but not in benign prostate tissues, suggesting its role in tumorigenesis [67]. Physical interaction of Trop2 and β-catenin was also observed in gastric cancer cell lines, where Trop2 is required for β-catenin nuclear accumulation and is associated with induction of EMT [66]. However, further studies are needed to identify the exact mechanisms that regulate processing, cellular localization, and posttranslational modifications of Trop2 and to clarify their effects on the biological function of Trop2.

Besides β-catenin and claudins, only a few Trop2-interacting partners have been identified so far, mostly affecting their signaling properties (Table 1). Further multi-omics and functional screenings are therefore necessary to reveal the whole spectrum of Trop2-interacting partners.

## 3. Trop2 in Cancer

In the last 40 years, the relation between both *TACSTD* gene family members, Trop2 and EpCAM, and cancer has been extensively studied. The functional role of EpCAM during tumorigenesis, tumor cell dissemination, its value as prognostic marker and therapeutic target has been nicely reviewed recently [78,79,80,81]. Similarly to EpCAM, the overexpression of Trop2 was observed in many types of carcinomas (Table 2). However, there are specific cancer types in which Trop2 is downregulated (Table 2). In general, overexpression of Trop2 often correlates with an unfavorable prognosis and increased risk of metastasis [66,82,83,84,85,86,87]. Nevertheless, there are exceptions to this trend and in some types of cancer, the downregulation is correlated with poor prognosis instead [88] (Table 2). Interestingly, prognostic value of Trop2 may also depend on its cellular localization within tumors. Ambrogi et al. reported that the membranous Trop2 is associated with worse survival, while the Trop2 intracellular retention is associated with better survival and less frequent disease relapse in breast cancer patients [89]. The importance of different Trop2 forms and cellular localizations in carcinoma prognosis, however, has to be further established.

High expression of Trop2 was also found in tumors of non-epithelial origin, such as melanomas [120], extranodal nasal NK/T cell lymphoma [121], gliomas and glioblastomas [68,122], and osteosarcomas [55]. Trop2 was also found to be overexpressed in pituitary adenomas [123].

### 3.1. Regulation of Trop2 Expression in Cancer

Although Trop2 is frequently overexpressed during tumorigenesis, genetic analyses revealed that point mutations and copy number variations in *TACSTD2* gene are rather rare in human tumors [16,124]; COSMIC database [124] accessed on 14 July 2020; Cancer Genome Atlas (https://www.cancer.gov/tcga) accessed on 14 July 2020). These data suggest that the overexpression of Trop2 in cancer does not arise from structural alterations of the gene itself but are rather results from deregulation at the transcriptional and posttranscriptional level. In an attempt to describe the Trop2-regulatory pathways in cancers, Guerra et al. identified a network of transcription factors that modulate Trop2 expression. This network includes TP63/TP53, ERG, GRHL1/Get-1, HNF1A/TCF-1, SPI1/PU.1, WT1, GLIS2, AIRE, FOXM1, and FOXP3 [125]. Moreover, subsequent studies identified the Trop2-regulatory function of CREB in breast cancer [126].

Signaling pathways that regulate Trop2 expression includes cyclooxygenase-2 and tumor necrosis factor-α in colon carcinoma cells [127,128], PTEN and lipoxygenase in prostate cells ([129,130], TGF-β in Langerhans cells [131], and fibroblast growth factor in embryonic lungs [132]. These pathways are often deregulated during tumorigenesis and may be, therefore, at least partially responsible for modulation of Trop2 expression in malignant cells. Interestingly, Trop2 expression can be as well induced by mechanical forces [17]. As mechanical stress arises during tumorigenesis [133], mechanotransduction could also represent an important factor in Trop2 regulation. This, however, remains to be proven experimentally.

Epigenetic factors were identified as important modulators of Trop2 expression in cancers, too. Hao et al. recently described circRNA—miR-488-3p—Trop2 regulatory loop that released Trop2 epigenetic silencing in head-and-neck squamous cell carcinoma cell lines [118]. Other miRNAs that target Trop2 were also identified in head-and-neck carcinoma [134] and urothelial bladder cancer [135]. Furthermore, we and others showed that Trop2 expression is regulated by methylation of *TACSTD2* gene promoter in various cancer cells [73,75,88,136,137]. Importantly, Lin et al. found hypermethylation of *TACSTD2* gene promoter in lung adenocarcinoma tissues and cell lines explaining low expression of Trop2 in this type of cancer [73]. Complex data from single-cell analyses may uncover the importance of all these factors as sources of Trop2 heterogeneity in various cancers.

### 3.2. Multiple Functions of Trop2 in Cancer

#### 3.2.1. Trop2 and EMT

Decreased adhesion and increased motility are important steps for successful metastatic colonization of secondary organs. In order to acquire the phenotype of mesenchymal cells with enhanced migratory capacity and invasiveness, polarized epithelial cells undergo an evolutionarily conserved transcriptional program known as EMT [138]. EMT is associated with a functional decrease in epithelial markers such as E-cadherin and upregulation of mesenchymal markers such as Vimentin, N-cadherin, and Fibronectin. Both EMT and its inversed process, known as the mesenchymal-to-epithelial transition (MET), are essential for the formation of metastases [139]. EMT is also hypothesized as one of the mechanisms leading to the generation of cancer cells with stem cell phenotype [139].

Our recent study found that the membrane expression of Trop2 positively correlates with E-cadherin expression and negatively with the mesenchymal gene signature in a wide panel of human and murine breast and prostate cancer cell lines, as well as human tumors [137]. Our findings suggest that in breast and prostate cancers, the surface Trop2 expression associates with the epithelial phenotype. Furthermore, we have shown that the Trop2 expression is suppressed either epigenetically through the DNA hypermethylation or EMT transcription factors, i.e., ZEB1 during EMT [137]. Similarly, the protein level of E-cadherin is significantly decreased in the absence of mTrop2 (murine Trop2) in immortalized murine keratinocytes [35]. The same study also shows that Ras-transformed keratinocytes deficient for Trop2 preferentially pass through EMT. In mTrop2-positive cells, the mTrop2 expression is lost during transformation associated with mesenchymal transdifferentiation. Moreover, *TACSTD2* mRNA levels are decreased in a subset of primary head-and-neck squamous cell carcinomas with features of EMT [35].

However, it should be noted that the association between Trop2 and mesenchymal phenotype was reported previously in other cancer types, suggesting that the link between Trop2 and EMT may be cancer type/cell context dependent. Li et al. described that the downregulation of Trop2 reduces the expression of Vimentin and upregulates E-cadherin in gallbladder cancer both in vitro and in vivo, while Trop2 overexpression has the opposite effects [56]. In this model, Trop2 is also involved in the PI3K/Akt pathway, since the downregulation of Trop2 inhibits Akt phosphorylation and increases the expression of PTEN, a negative regulator of Akt activity [56]. PI3K/Akt pathway is well-known to induce EMT [140]. The findings by Li et al. are supported by previously published studies, which have also found that high expression of Trop2 is associated with reduced expression of E-cadherin in cervical cancer cell lines [62], gallbladder cancer [105], and gastric cancer cell lines [66]. In nasopharyngeal carcinoma cells, Trop2 promotes EMT through the activation of NF-κB [54]. Inactivation of the NF-κB pathway in these cells attenuates Trop2-induced invasion and EMT. Overexpression of Trop2 leads to an increased N-cadherin, Vimentin, and Twist as well as decreased E-cadherin, while Trop2 downregulation has the opposite effect. In gastric cancer cell lines, Trop2 binds to β-catenin and promotes its nuclear translocation and accumulation, increasing its transcription activity and contributing to the EMT [66]. Inhibition of EMT by Trop2 silencing has been observed recently in endometrial cancer cells as well [57].

Overall, the results of studies linking Trop2 with EMT are contradictory, and the precise role and significance of Trop2 in this process remain to be elucidated. We believe that understanding the functional consequences of EMT-related intratumoral heterogeneity in Trop2 expression is of great importance, as it may significantly affect the response to therapies targeting Trop2-expressing cells.

#### 3.2.2. Trop2 and Cell Proliferation

In many tumor types, Trop2 stimulates proliferation and cellular growth. In cervical cancer cells, Trop2 promotes proliferation by regulating the ERK signaling pathway [62]. Down-regulation of Trop2 results in decreased cyclin D1, cyclin E, cyclin-dependent kinases (CDK)2, and CDK4 expression, as well as in an increased p27, an inhibitor of CDKs. Similarly, in human bladder cancer cell lines, Trop2 overexpression suppresses the protein levels of p27 and induces expression of cyclin E1 [141]. Interestingly, Trop2 overexpression combined with curcumin treatment partially abrogates these effects, and it seems that curcumin exhibits some of its anti-tumor activity by regulating Trop2 expression. In murine pancreas adenocarcinoma cell line, mTrop2 also increases levels of phosphorylated ERK1/2, which leads to an increase in proliferation activity in low serum conditions [60].

Overexpression of ectopic Trop2 in a large panel of immortalized and transformed cell lines stimulates cell growth, associated with higher proportions of cells in the S phase of the cell cycle [16]. In the same study, Trop2 potentiates tumor growth in vivo proportionally to the Trop2 expression levels. Trop2 also enhances the capacity of colon cancer cells for anchorage-independent growth [142]. In glioblastoma cells, Trop2 promotes growth and dissemination by the activation of the JAK2/STAT3 signaling pathway [68] and attenuates the expression of molecules downstream of this signaling pathway such as cyclin D1, survivin, matrix metalloproteinase 2 (MMP2), and vascular endothelial factor (VEGF). Furthermore, knockdown of Trop2 in glioblastoma cells leads to the inhibition of JAK/STAT phosphorylation.

On the other hand, the ability of Trop2 to suppress cell proliferation was also reported. In lung adenocarcinoma, Trop2 expression is often downregulated [73]. While the increased Trop2 expression in one lung cancer cell line leads to the suppression of cell proliferation, the silencing of Trop2 in another cell line promotes it. Trop2 knockdown also significantly stimulates cell proliferation and migration in cholangiocarcinoma cell lines and is associated with increased ERK phosphorylation [75]. Furthermore, knockdown of Trop2 in the cervical cancer cells promotes cell proliferation, migration, and invasion capabilities in vitro and enhances tumor growth capability in vivo [77]. In the MCF7 breast cancer cell line, knockdown of Trop2 slightly increases proliferation when compared to control cells [143]. The above-described findings suggest that the role of Trop2 in regulating proliferation is a complex, cell type-, and organ-specific phenomenon.

#### 3.2.3. Trop2 and Cell Adhesion and Migration

The ability of Trop2 to promote migration and invasion of cancer cells was described in several types of tumors, but different mechanisms of Trop2 involvement were suggested. In prostate cancer cells, Trop2 was shown to promote cell motility by inhibition of cell adhesion to the extracellular matrix glycoprotein Fibronectin [71]. This is achieved by a complex mechanism comprising direct interaction of Trop2 with α5β1 Integrin complex and Talin, their relocalization from focal adhesions to the leading edges, RACK1 translocation to cell membrane, Src and FAK activation resulting in a faster turnover of adhesive structures and destabilization of the α5β1Integrin complex bond to Fibronectin (Figure 2) [71]. Interestingly, Trop2-positive exosomes were found to promote migration of Trop2-negative prostate cancer cells on Fibronectin by a yet unknown mechanism [69]. In thyroid cancer cells, Trop2 stimulates the expression of MMP2 through an MAPK ERK/JNK signaling pathway and enhances the invasion of these cells [61]. As briefly mentioned above, the migratory capacity of colorectal carcinoma cells is dependent on phosphorylation of serine 322 of Trop2, resulting in Claudin 7 relocalization. Blocking of this phosphorylation site decreases such migration, and cells expressing phospho-mimetic Trop2 exhibit higher migratory capacity [13].

#### 3.2.4. Trop2 and Drug Resistance

It is not surprising that Trop2 appears to have a dual function in the regulation of cancer cell survival and drug resistance as well. Overexpression of Trop2 in cervical cancer cell lines inhibits apoptosis by increasing the expression of Bcl-2 and decreasing the expression of Bax [62]. Similarly, the downregulation of Trop2 in ovarian carcinoma cells decreases Bcl-2 and increases Bax expression [144]. In bladder cancer cell lines, transient silencing of Trop2 leads to an increased apoptosis and sensitizes cells to curcumin treatment, while Trop2 overexpression reduces the frequency of apoptotic cells and abrogates curcumin-induced cell death [141]. Inhibition of Trop2 significantly increases apoptosis of non-small cell lung carcinoma (NSCLC) cells [99] and overexpression of Trop2 decreases apoptosis in oral squamous cell carcinoma cell lines [58]. In prostate cancer xenografts, Trop2 was upregulated in relapsed tumors after flutamide and docetaxel treatment and Trop2 enhanced recovery of androgen-sensitive, but not androgen-resistant cells, after exposure to docetaxel [145]. Silencing Trop2 using shRNA resulted in increased sensitivity to cisplatin in lung and cisplatin/5-fluorouracil in gastric cancer cells in vitro and in vivo [146,147].

In contrast to these findings, Trop2-overexpressing cervical cancer cells are more sensitive to cisplatin-induced apoptosis, while such cells with silenced Trop2 expression are more resistant [77]. Similarly, Trop2 silencing reduced the sensitivity of transformed keratinocytes to gemcitabine [64].

As expected, modulation of Trop2 signaling through the epidermal growth factor receptor 3 (ErbB3), insulin-like growth factor 1 receptor (IGF-1R), and Akt pathways (see Chapter 2.2 and 3.2.6) reflects the observed association between Trop2 expression and resistance to EGFR inhibitor gefinitib in head-and-neck squamous cell carcinoma [148], altered response of Trop2-overexpressing cells to IGF-1R inhibitor AG-1024 in HeLa cells [77], and reduced response of Trop2 shRNA transfected breast cancer cells to allosteric Akt inhibitors [59].

#### 3.2.5. Other Functions in Cancer

Furthermore, several studies imply a possible role of Trop2 in angiogenesis. In glioblastoma cells, Trop2 overexpression upregulates VEGF levels, and accordingly, its downregulation leads to decreased VEGF expression [68]. VEGF is a potent angiogenic factor that promotes the growth of blood vessels, which allows tumor growth and dissemination. In gliomas [122] and hilar cholangiocarcinomas [86], Trop2 expression positively correlates with microvessel density.

It was also found that in Trop2 and seven other genes, encoding barrier molecules are highly expressed in a subset of metastatic melanomas and ovarian carcinomas, and their expression is associated with a lack of immune gene signatures and worse prognosis [120]. This subset of tumors with high expression of barrier molecules is usually not infiltrated by T cells. Such T cell infiltration of tumors is associated with an improved prognosis in different types of cancer [120].

A recent study shows that Trop2 induces neuroendocrine phenotype of prostate cancer, and overexpression of Trop2 leads to the significant increase in Poly(ADP-Ribose) polymerase 1 (PARP1) [84], an enzyme critical for DNA repair regulation, replication, transcription, and chromatin remodeling [149]. Trop2 overexpression results in increased DNA replication and accumulation of DNA damage even though PARP1 and other DNA repair proteins are upregulated in these cells [84]. It was proposed that Trop2 regulates PARP1 through the upregulation of c-MYC, but this remains to be elucidated. In vivo, inhibition of PARP1 leads to the reversion of neuroendocrine phenotype and a decrease in tumor growth and metastasis.

#### 3.2.6. Trop2 as Tumor Suppressor (Until It Is Not)

It is broadly accepted that in a wide spectrum of cancers high Trop2 expression promotes tumor growth [16] and positively correlates with metastasis and poor prognosis, suggesting that Trop2 acts as a putative oncogene. However, opposite findings were reported, showing that in specific cases Trop2 acts as a tumor suppressor.

Low expression of Trop2 in lung adenocarcinomas was attributed to the DNA hypermethylation in the *TACSTD2* promoter region or by the loss of heterozygosity [73]. In lung adenocarcinoma cell lines, Trop2 interacts with insulin-like growth factor 1 (IGF-1) and prevents its binding to the IGF-1R. This further blocks the Akt and ERK kinases activation, β-catenin, and Slug expression and reduces cell proliferation. Accordingly, loss of Trop2 expression results in the stimulation of cell proliferation and tumor growth in vivo. A study by Pak et al. shows that Trop2 overexpression is associated with better survival in patients with NSCLC [74]. However, another study reported an association between high Trop2 expression, metastasis, and poor prognosis in another NSCLC cohort. Furthermore, Trop2 overexpression in lung adenocarcinoma cell lines stimulates cell proliferation, migration, and invasion, whereas knockdown of Trop2 induced apoptosis [99].

Trop2 also exhibits tumor suppressive functions in cervical cancer cells, where it similarly inhibits the activation of IGF-1R and anaplastic lymphoma kinase (ALK), possibly through binding to their ligands IGF-1 and midkine (MDK) [77]. Another such example is the reduced or completely lost expression of Trop2 in the mesenchymal subtype of squamous head-and-neck carcinoma [35]. Decreased Trop2 expression leads to increased phosphorylation of the ErbB3 receptor, also known as HER3, in this cancer type. As Trop2 interacts with neuregulin-1 (NRG-1), a ligand of ErbB3, inactivation of Trop2 increases the concentration of NRG-1 on the cell surface, which is then cleaved by metalloproteinase TACE [65]. The released extracellular domain of NRG-1 then activates ErbB3, promoting cell proliferation and tumor growth (Figure 2). Squamous cell carcinomas are characterized by a gradual loss of Trop2 during progression, inversely proportional to ErbB3 [64]. Interestingly, resistance to anti-ErbB3 treatment in head-and-neck squamous cell carcinoma is associated with increased Trop2 protein expression [63]. Co-treatment with both anti-ErbB3 and anti-Trop2 antibodies leads to a greater anti-tumor response than either antibody alone.

Moreover, decreased expression of Trop2 has been found in liver fluke-associated cholangiocarcinomas [75] and hepatocellular carcinomas [49,88]. Trop2 downregulation is in both cases epigenetically controlled by hypermethylation of *TACSTD2* promoter. Low Trop2 expression associates with poor overall survival, invasion, and poor differentiation in hepatocellular carcinomas [88], but not in cholangiocarcinomas [75]. Similarly, our study shows that in lymph-node-positive breast cancer and prostate cancer patients, low expression of *TACSTD2* mRNA associates with worse prognosis [137].

Interestingly, exposure of mTrop2^−/−^Arf^−/−^ mice to 7, 12-dimethylbenzanthracene and 12-O-tetradecanoylphorbol-13-acetate (DMBA-TPA) results in the formation of skin carcinomas, whereas none of the mTrop2^+/+^:Arf^−/−^ mice developed tumors [35]. Immortalized keratinocytes derived from mTrop2^−/−^Arf^−/−^ exhibited enhanced proliferative and migratory capacity when compared to mTrop2^+/+^Arf^−/−^.

It again seems that there is more than one possible interpretation of such conflicting findings. The precise role of Trop2 likely depends on the wider genetic context of a particular cancer cell. A phenomenon commonly known as non-oncogene addiction may further explain these contradictions [5]. In this situation, tumor cells are hyper-dependent upon non-oncogenic genes for survival within the physiologically stressful microenvironment. Moreover, the results of the reviewed studies also suggest the possibility that the role of Trop2 changes gradually, from early tumorigenesis, where it acts as a tumor suppressor, to later stages, where its reactivation induces metastasis and associates with the worse prognosis. Unlike the “genetic context hypothesis”, this gradual increase in Trop2 explains its widespread expression in cancer, regardless of its dual role in many related processes. This speculation, however, requires further testing in relevant systems. Another question that remains yet unanswered is the impact of various Trop2 forms and their subcellular localization on cancer cell behavior. This was addressed by only a very limited number of studies, although the signaling properties, oncogenic or tumor suppressive role, and the prognostic value of these different forms in tumors deserve further thorough investigation.

### 3.3. Trop2 in Cancer Therapy

Due to its frequent overexpression in tumors, Trop2 seems to be an exceptionally promising candidate for immunotherapeutic strategies. The murine monoclonal antibody RS7-3G11 has been raised against human NSCLC cells [150]. This antibody showed pan-carcinoma specificity including breast, colon, lung, renal, stomach, bladder, breast, ovary, uterus, and prostate [151]. Subsequently, Trop2 was identified as its antigen [10]. This clone promoted antibody-dependent cell-mediated cytotoxicity in vitro in ovarian [102], cervical [106], uterine [108], and endometrial carcinoma cells [111]. Radiolabeled RS7 was efficient in targeting and killing human cancer xenografts in immunocompromised mice [152,153,154]. This antibody was rapidly internalized from the surface of cancer cells [151,155], providing an opportunity for targeted delivery of cytotoxic compounds to Trop2-expressing cancers. This approach was tested with mutant Rap toxin conjugated with a humanized RS7 (hRS7) antibody, and these experimental conjugates suppressed the growth of epithelial cancer cell lines and xenografts [156,157].

The antibody–drug conjugate IMMU-132, also known as sacituzumab govitecan, was formed by linking the hRS7 antibody to topoisomerase I inhibitor SN-38 [158]. SN-38 is an active metabolite of irinotecan, the chemotherapeutic agent often used for clinical management of colorectal carcinomas [159]. The development of this conjugate and the results of preclinical and clinical studies in various cancer types have been reviewed recently [160,161]. These studies demonstrated a significant clinical response, good pharmacokinetic profile, no immunogenicity, and manageable toxicity of sacituzumab govitecan in heavily pretreated metastatic cancers, especially in triple-negative breast cancer (TNBC) [162,163,164,165,166,167,168,169,170,171]. Based on these results, sacituzumab govitecan received the Breakthrough Therapy Designation from the U.S. Food and Drug Administration (FDA) in 2016 [172], and during the preparation of this review, the FDA accelerated its approval for the treatment of adult patients with metastatic TNBC who have received at least two prior therapies for metastatic disease [173].

Unfortunately, clinical studies have not provided conclusive evidence that Trop2 levels predicted responsiveness to sacituzumab govitecan, possibly because of the high frequency of strong positive specimens [161]. Further clinical studies are therefore needed to select patients for this type of therapy, including those with less advanced disease, to investigate effective therapeutic combinations and to find suitable predictive markers. Clinical trials currently testing sacituzumab govitecan are summarized in Table 3.

Besides sacituzumab govitecan, several other Trop2-targeting immunotherapies are being developed and tested. A hRS7 conjugate with auristatin derivative, a microtubule polymerization inhibitor, was tested in preclinical studies and phase I clinical study in solid metastatic tumors [174,175]. However, severe adverse effects and only modest anti-tumor activity were observed [174].

In 2014, a human Fab antibody against the Trop2 extracellular domain was isolated from a phage library [176]. Trop2 Fab antibody itself induces apoptosis and inhibits proliferation and migration of breast carcinoma cells in vitro, and the growth of breast cancer xenografts in vivo. It also leads to elevated Bax expression and reduced Bcl-2 expression, both in vitro and in vivo. Two years later, Mao et al. prepared Trop2Fab-DOX conjugate that specifically binds to pancreatic cancer cells expressing Trop2, inhibiting their proliferation and migration in vitro and reducing the tumor growth in vivo [177].

Further development of Trop2-targeting therapies involves nanoparticles conjugated with anti-Trop2 antibody [178]. These nanoparticles consist of carboxymethyl dextran derivatives with bioreducible disulfide bonds loaded with doxorubicin. After binding to Trop2, nanoparticles enter the cells by endocytosis where doxorubicin is released. In vitro experiments show that nanoparticles are selectively taken up by Trop2-expressing TNBC cell lines and exhibit higher toxicity than control nanoparticles lacking the disulfide bond or anti-Trop2 antibody.

Other approaches tested includes Trop2 bispecific antibodies that target T cells to tumors [179], immunization with virus-like particles incorporating Trop2 [180,181] and anti-Trop2-based photothermal therapy [182].

## 4. Conclusions

Studies in the past decade showed that Trop2 is a critical regulator of multiple essential processes involved in carcinogenesis and cancer progression. Even though initially identified as an oncogene [142], it turns out that the role of Trop2 is not that straightforward. Recent data suggest that Trop2 acts both as an oncogene and tumor suppressor. The exact reason for these opposite observations remains to be determined. The therapeutic potential of Trop2 has been pursued in several successful clinical trials. Independently of its function, the simple fact that Trop2 is overexpressed in most cancers, while healthy tissues express it only sporadically, making it an incredibly promising target for cancer-specific delivery of cytotoxic agents. Such a strategy is already utilized by several drugs, while others are being developed. However, it should be noted that Trop2-targeting therapy may spare the Trop2-negative cells within heterogeneous tumors. Further pre-clinical and clinical studies are necessary to clarify the fate and properties of this cell population in response and resistance to Trop2-targeting therapies.

## Figures and Tables

**Table 1:** List of proteins interacting with Trop2 and cellular functions of these interactions.

Trop2-Interacting Partner	Function	Reference
β-catenin	activation of β-catenin signaling	[66,67]
Claudin 1 and 7	facilitation of proper localization	[46]
Occludin	facilitation of proper localization	[49]
α5β1 integrin/Talin complex	relocalization from focal adhesions to leading edges, rearrangement of focal adhesions sites through displacement of FAK	[69,70]
IGF-1	inhibition of IGF-1R signaling	[73,77]
MDK	inhibition of ALK signaling	[77]
NRG-1	inhibition of ErbB3 activation	[65]

**Table 2:** Table list alterations in Trop2 expression and effects on prognosis in different types of carcinomas.

**Trop2 Overexpression**					
**Type of Carcinoma**	**Detection Method**	**Number of Subjects**	**Protein Location**	**Prognosis**	**Reference**
gastric	IHC	104	membrane	decreased overall and disease-free survival (in patients with intestinal-type carcinoma and lymph-node-positive patients)	[85]
	IHC	830	membrane, cytoplasm	decreased overall and disease-free survival	[90]
	qRT-PCR	41 pairs	-	-	
	IHC	330	membrane, cytoplasm	decreased overall survival (in patients with Trop2+/vimentin+ expression)	[66]
	IHC	844	membrane	N/A	[91]
	ICC	192	-	-	
thyroid	IHC	124	membrane, cytoplasm	-	[61]
	qRT-PCR	18 pairs	-	-	
	RNA seq	59 pairs	-	-	
papillary thyroid	ICC	60	-	-	[92]
	IHC	94	membrane, cytoplasm	-	
	IHC	433	membrane	-	[93]
	IHC	468	membrane	-	[94]
colorectal	IHC	620	-	decreased overall survival and increased disease recurrence	[95]
	IHC	34	-	-	[96]
	qRT-PCR	74 pairs	-	decreased overall survival	
non-small cell lung	IHC	104	membrane, nucleus, cytoplasm	decreased overall survival (in patients with adenocarcinoma)	[97]
	IHC	334	membrane	decreased overall survival (in patients with adenocarcinoma)	[98]
	IHC	164	membrane	better overall and disease-free survival (in patients with adenocarcinoma)	[74]
lung adenocarcinoma	IHC	130	membrane, cytoplasm	decreased overall survival (in patients with non-lepidic type tumors)	[76]
	IHC	68	membrane, cytoplasm	decreased overall survival	[99]
	qRT-PCR	20 pairs	-	-	
breast	IHC	702	membrane, cytoplasm	decreased overall survival associated with membrane Trop2, intracellular Trop2 associated with better overall survival and disease recurrence	[89]
	IHC	354		decreased overall survival (in patients with Trop2+/E-cadherin- expression)	[87]
	qRT-PCR	20 pairs	-	-	
ductal breast	IHC	152	membrane	decreased overall survival	[100]
	qRTPCR	15 pairs	-	-	
pancreatic	IHC	207	membrane	decreased overall survival and shortened progression-free survival in patients who underwent surgery with curative intent	[82]
ovarian	IHC	117	membrane, cytoplasm	decreased overall and progression-free survival	[101]
	qRT-PCR	128	-	N/A	
	IHC	55	membrane	-	[102]
	IHC	90	membrane, cytoplasm	-	[103]
prostate	IHC	339	membrane, cytoplasm	-	[84]
	IHC	148	membrane	-	[69]
bladder	IHC	40	membrane, cytoplasm	-	[104]
gallbladder	IHC	93	membrane, cytoplasm	decreased overall survival	[105]
	IHC	105	membrane, cytoplasm	decreased overall survival	[56]
	qRT-PCR	56	-	-	
	WB	10	-	-	
cervical	IHC	160	membrane	decreased overall and progression-free survival	[62]
	IHC	13	membrane	-	[106]
	IHC	147	membrane, cytoplasm	-	[107]
uterine serous papillary	IHC	28	membrane	-	[108]
	IHC	104	membrane, cytoplasm	-	[109]
uterine and ovarian carcinosarcomas	IHC	10	membrane, cytoplasm	-	[110]
endometrial	IHC	163	membrane	-	[111]
	IHC	118	membrane	decreased disease-free survival	[112]
nasopharyngeal	qRT-PCR	37	-	-	[54]
	IHC	58	membrane, cytoplasm	decreased overall and disease-free survival	[113]
hilar cholangiocarcinoma	IHC	70	membrane	decreased overall survival	[86]
	qRT-PCR	56 pairs	-	-	
oral squamous cell	IHC	90	membrane	decreased overall survival	[83]
	IHC	443	membrane, cytoplasm	decreased overall and disease-free survival	[114]
	qRT-PCR	93	-	-	
	IHC	320	membrane, cytoplasm	decreased overall survival (in patients with Trop2+/P16- expression)	[115]
	IHC	108	cytoplasm	decreased overall survival	[116]
esophageal squamous cell	IHC	55	membrane, cytoplasm	-	[117]
head-and-neck squamous cell	IHC	42	membrane	decreased overall and disease-free survival	[118]
laryngeal squamous cell	IHC	137	membrane, cytoplasm	decreased overall and disease-free survival	[119]
	qRT-PCR	15 pairs	-	-	
**Trop2 Downregulation**					
**Type of Carcinoma**	**Detection Method**	**Number of Subjects**	**Protein Location**	**Prognosis**	**Reference**
liver fluke-associated cholangiocarcinoma	IHC	85	membrane, cytoplasm	N/A	[75]
lung adenocarcinoma	IHC	55 pairs	membrane	-	[73]
	LOH analysis	119	-	-	
hepatocellular	qRT-PCR	205 pairs	-	decreased overall survival	[88]
	RNA seq	3 pairs	-	-	
squamous cell carcinoma of cervix	IHC	79	membrane, cytoplasm	-	[64]
squamous cell carcinoma of head and neck	IHC	5	membrane, cytoplasm	-	
squamous cell carcinoma of esophagus	IHC	6	membrane, cytoplasm	-	

Only patient-related data from clinical studies are included. IHC stands for immunohistochemistry, ICC stands for immunocytochemistry, LOH stands for loss of heterozygosity, WB stands for Western blot, qRT-PCR stands for quantitative reverse-transcription PCR, N/A stands for no association with prognosis, - means association with prognosis was not studied.

**Table 3:** List of clinical trials that test the efficacy of sacituzumab govitecan alone or in combined settings.

ClinicalTrials.gov Identifier	Disease	Combined Therapy	Phase
NCT03964727	Metastatic NSCLC, head-and-neck squamous cell carcinoma, endometrial cancer	-	2
NCT03725761	Metastatic castration-resistant prostate cancer	-	2
NCT04230109	Invasive localized TNBC	-	2
NCT01631552	Advanced epithelial cancers	-	1/2
NCT03547973	Metastatic urothelial carcinoma	Pembrolizumab	2
NCT03901339	HR+/HER2- metastatic breast cancer	-	3
NCT02574455	Refractory/relapsed metastatic TNBC	-	3
NCT04039230	Metastatic breast cancer	Talazoparib	1/2
NCT03995706	Glioblastoma, metastatic brain tumors	-	early 1
NCT03992131	Metastatic solid tumors—ovarian cancer, TNBC, urothelial carcinoma	Rucaparib	1/2
NCT04251416	Persistent of recurrent endometrial carcinoma	-	2
NCT04319198	Metastatic solid tumor	-	3
NCT03424005	Metastatic or inoperable locally advanced TNBC	Atezolizumab	1/2
NCT04454437	Metastatic TNBC	-	2
NCT04468061	Metastatic TNBC	Pembrolizumab	2
NCT04448886	Metastatic HR+/HER2- breast cancer	Pembrolizumab	2
NCT03337698	Metastatic NSCLC	Atezolizumab	1/2
NCT04559230	Glioblastoma	-	2
NCT04527991	Metastatic or locally advanced unresectable urothelial cancer	-	3

The database was accessed on 6 October 2020.
